# Supplementary material for: Variations in the glucosinolates of the individual edible parts of three stem mustards (Brassica juncea)
Source: R Soc Open Sci. 2019 Feb 20;6(2):182054. doi: 10.1098/rsos.182054 (PMC6408409; doi:10.1098/rsos.182054)
Supplement: Supplemental Fig. 1 [file rsos182054supp1.docx]

*Royal Society Open Science*

Variations in the glucosinolates of the individual edible parts of three stem mustards (*Brassica juncea*)

Bo Sun^1,‡^, Yu-Xiao Tian^1,‡^, Qing Chen^1^, Yong Zhang^1^, Ya Luo^1^, Yan Wang^2^, Meng-Yao Li^1^, Rong-Gao Gong^1^, Xiao-Rong Wang^2^, Fen Zhang^1,^* and Hao-Ru Tang^1,2,^*

^1^College of Horticulture, Sichuan Agricultural University, Chengdu 611130, China

^2^Institute of Pomology and Olericulture, Sichuan Agricultural University, Chengdu 611130, China

‡ These authors contributed equally to this work.

Authors for correspondence:

Fen Zhang

email: [zhangf_12@163.com](mailto:zhangf_12@163.com);

Hao-Ru Tang

email: [htang@sicau.edu.cn](mailto:htang@sicau.edu.cn)

Supplemental Figure 1


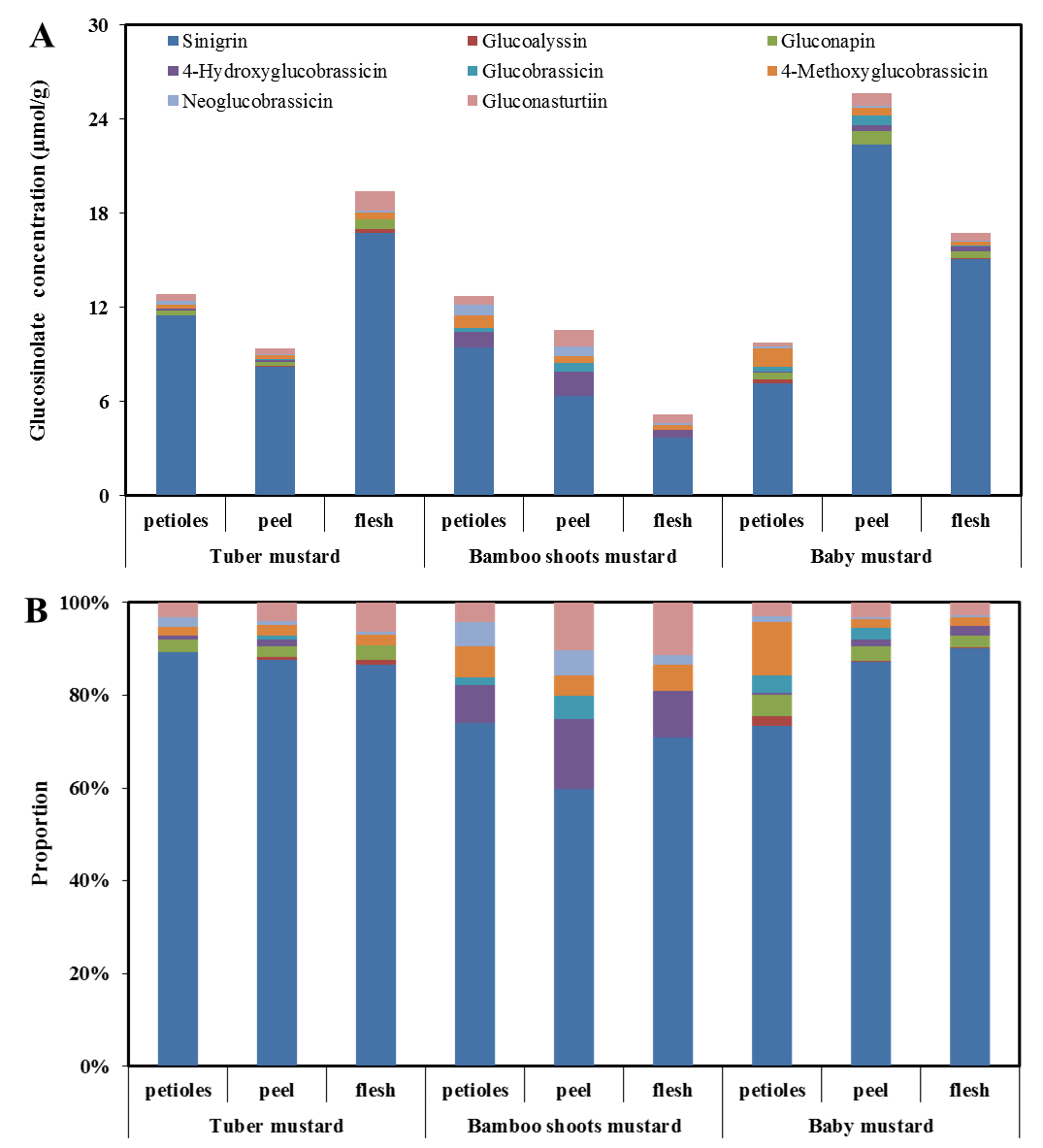


Supplemental Fig. 1 The concentration and proportion of individual glucosinolates in different tissues of the three stem mustards. A: glucosinolate concentration. B: proportion.
